# Greater visceral fat mass accumulation with high alcohol consumption

**DOI:** 10.1038/s41366-026-02030-5

**Published:** 2026-02-25

**Authors:** Joel Chesters, Matt J. Neville, Fredrik Karpe

**Affiliations:** 1 Oxford Centre for Diabetes, Endocrinology and Metabolism, Radcliffe Department of Medicine, University of Oxford, Churchill Hospital, Oxford, UK; 2 NIHR Oxford Biomedical Research Centre, OUH Trust, Oxford, UK

**Keywords:** Obesity, Fat metabolism, Obesity, Nutrition, Epidemiology

## Abstract

Regular alcohol consumption is commonly perceived to contribute to abdominal adiposity, particularly visceral fat mass (VFM). Observational studies in this area have either been small or used imprecise measurement methods of VFM (e.g., waist circumference). More precise methods, such as dual-energy x-ray absorptiometry (DXA), have not been used at scale to explore the association between alcohol consumption and VFM. We investigated the relationship between alcohol consumption and VFM in a population-based cohort of *n* = 5 761 men and women from the Oxford Biobank who underwent a DXA-scan and provided information on regular alcohol consumption using a structured questionnaire. After adjustment for key confounders - age, smoking, height, physical activity, socio-economic status, and total fat mass (TFM) - alcohol consumption remained dose-dependently associated with VFM, in males (ß = 1.104 (1.040–1.167), *p* < 0.001) and females (ß = 1.102 (1.029–1.180), *p* = 0.006). The mean VFM percentage (%VFM) in the highest alcohol consumption quartile was over 10% greater, relative to the mean %VFM in the adjacent lower quartile, in both males (median 24 vs 12 units/week) and females (median 14 vs 7 units/week). Elevated VFM among heavier drinkers may contribute to poorer cardiovascular and metabolic health and is relevant in the search for mechanisms of regional fat repositioning.

## Introduction

Regular alcohol consumption is commonly assumed to promote uneven truncal fat accumulation, colloquially referred to as a “beer belly”. Enhanced imaging techniques (e.g., MRI, CT, or DXA) are required to verify whether this abdominal fat is superficial or intra-abdominal/visceral fat mass (VFM).

Alcohol is the second-most energy-dense ‘macronutrient’ (after fat) [1], contributing substantially to daily caloric intake in heavy drinkers. Strong associations between alcohol consumption and measures of total fat have been reported [2–5], yet its effect on specific fat depots remains unclear, limiting explanations for the regional fat accumulation attributed to heavy drinking.

Ethanol, or its metabolites, may directly influence adipose tissue by both inhibiting lipolysis, and providing substrates for de novo lipogenesis [1, 6]. Acetaldehyde, the primary metabolite of ethanol, may also stimulate the hypothalamic-pituitary-adrenal (HPA) axis, promoting a pseudo-Cushing’s syndrome, that evokes truncal adiposity [7, 8]. In extreme cases of heavy drinking, multiple symmetric lipomatosis (Madelung’s disease) can be seen, supporting an alcohol-specific impact on regional fat distribution [9]. Finally, recent evidence suggests that acetate, generated by hepatic ethanol oxidation, may induce histone acetylation in distant tissues, profoundly altering global transcriptional control [10], though this mechanism has not been explored in adipose tissue.

Several studies examining alcohol and body composition have used imprecise measures of VFM. Large-scale observational studies often rely on conventional anthropometrics (e.g., waist circumference, waist-to-hip ratio) [2–4], which cannot accurately distinguish between visceral and subcutaneous fat. Furthermore, the strong trend of increasing BMI with greater alcohol consumption tends to weaken associations after BMI adjustment [4].

Most studies employing more precise imaging methods (CT, MRI or DXA) have been small [11], or focused on heavy drinkers/alcoholics [12, 13]. The larger studies commonly faced similar limitations, such as exclusively studying one sex, limiting their generalisability to the wider population [5, 14].

To overcome the issues of sample size, population representation, and precise VFM quantification, we assessed the relationship between alcohol consumption and VFM in the Oxford BioBank, which is a population-based cohort (*n* = 5761) with DXA-measured VFM.

## Subjects and methods

### Participants

This study analyses a cohort of men and women from the Oxford Biobank [15], including 10% of participants with Type 2 diabetes (T2D). This ensures that the proportion of people with T2D closely represents the general UK population (nearly 8% population prevalence [16]). The cohort protocol has ethical approval (REC 23/SC/0411).

### Measurement of alcohol consumption and VFM

Information on alcohol consumption was collected via a structured questionnaire as part of the Oxford Biobank screening visit. Participants were asked to recall typical weekly alcohol consumption in standardised units from stated volumes of alcoholic beverages (1 unit = 8 g of pure alcohol, i.e., half a pint of beer/cider, 1 small glass of wine, 1 pub measure of spirits). The results were expressed as total weekly units. Total fat mass (TFM) and VFM were measured using a DXA scan (GE Lunar iDXA, Encore software [15]), which correlates well with CT-derived measurements of fat mass [17].

### Statistical analysis

Analyses were stratified by sex and drinking status (“non-drinkers” and “drinkers”). We sought to examine if there was a dose-dependent relationship between alcohol consumption and VFM. Participants reporting zero alcohol intake formed a separate group, as non-drinkers are known to be a heterogenous group that may include lifelong abstainers as well as former heavy drinkers [18].

“Drinkers” were stratified into sex-specific alcohol consumption quartiles. A one-way-ANOVA (post-hoc Scheffé test) was used to test for significant differences in VFM between the quartiles.

To assess whether higher alcohol consumption is associated with a greater proportion of visceral relative to total fat, we calculated percentage VFM (%VFM = VFM/TFM x 100). Analysis of covariance (ANCOVA) tested for differences in the %VFM across alcohol quartiles, adjusting for confounders, with Bonferroni correction for multiple comparisons. Confounders used in the statistical models were age, smoking, height (standardised stadiometer measurement), physical activity level (sedentary, moderately active, active, or fit), and socio-economic status, derived from deciles of the postcode-associated index of multiple deprivation [19]. These confounders were selected to account for maximal variance in VFM while avoiding multicollinearity.

In a supplementary analysis, the TFM-VFM relationship was plotted for each alcohol group (including non-drinkers) and an interaction model (VFM ~ TFM x alcohol quartile) tested whether the slopes differed between groups.

Alcohol consumption and VFM were also analysed as continuous variables in multivariate regression, adjusted for the same confounders with added TFM to account for total adiposity, as this model examined absolute rather than proportional VFM. Log_10_-transformations were applied to satisfy normality assumptions and regression coefficients were back-transformed for interpretation. Additional analyses examined waist circumference as an alternative measure of abdominal adiposity, with BMI replacing TFM in this model.

## Results

We considered data from 5932 males and females, aged 25–75 years. After excluding 171 individuals with incomplete alcohol or anthropometric data, 5761 participants (42.8% male and 57.2% female) were included in the analysis. Non-drinkers (*n* = 810) formed one group, and the remaining participants (drinkers, *n* = 4 951) were stratified into equally-sized, sex-specific quartiles of weekly consumed units of alcohol: Q1: 1–4 (male) and 1-2 (female), Q2: 4–10 (male) and 2–5 (female), Q3: 10–17 (male) and 5–10 (female), Q4: 17–98 (male) and 10–50 (female). Descriptive statistics are presented in Table 1.

**Table 1:** Descriptive statistics separated by sex and stratified into alcohol consumption groups (mean ± SD).

Measurement	Male						Female					
	Non-drinkers	Q1	Q2	Q3	Q4	Total	Non-drinkers	Q1	Q2	Q3	Q4	Total
*n*	248	555	555	555	555	2468	562	683	683	683	682	3293
Age (years)	42.0 ± 6.4	42.5 ± 8.8	43.2 ± 8.8	43.6 ± 8.7	44.8 ± 8.7	43.4 ± 8.6	41.6 ± 6.8	41.7 ± 7.9	42.0 ± 7.6	42.8 ± 7.5	45.4 ± 8.2	42.7 ± 7.8
Alcohol Consumption (units/week)	0 ± 0	2.1 ± 1.0	6.2 ± 1.8	12.4 ± 2.3	27.0 ± 10.4	10.7 ± 10.9	0 ± 0	1.4 ± 0.5	3.6 ± 0.9	7.2 ± 1.6	15.4 ± 5.8	5.7 ± 6.2
Waist Circumference (cm)	93.9 ± 13.2	91.0 ± 11.2	92.5 ± 11.0	93.0 ± 10.2	95.4 ± 10.5	93.1 ± 11.1	85.3 ± 14.1	82.5 ± 12.7	81.0 ± 11.4	80.6 ± 10.7	83.5 ± 11.6	82.5 ± 12.2
Hip Circumference (cm)	102.0 ± 8.6	100.7 ± 7.1	101.4 ± 7.2	101.9 ± 7.1	102.6 ± 6.9	101.7 ± 7.3	103.7 ± 11.4	101.8 ± 10.1	100.6 ± 8.9	100.4 ± 8.6	101.4 ± 9.1	101.5 ± 9.7
BMI	27.1 ± 4.8	26.0 ± 4.0	26.3 ± 3.9	26.5 ± 3.7	27.3 ± 3.9	26.6 ± 4.0	26.7 ± 5.9	25.4 ± 4.9	24.7 ± 4.5	24.7 ± 4.5	25.3 ± 4.4	25.3 ± 4.9
Total Fat Mass (kg)	23.8 ± 10.9	21.6 ± 8.9	22.6 ± 8.5	22.9 ± 8.3	24.5 ± 8.4	23.0 ± 8.9	27.4 ± 11.8	24.9 ± 10.1	23.8 ± 9.1	23.3 ± 8.9	24.7 ± 9.3	24.7 ± 9.9
Visceral Fat Mass (kg)	1.21 ± 0.96	0.97 ± 0.80	1.09 ± 0.78	1.12 ± 0.75	1.36 ± 0.82	1.14 ± 0.82	0.54 ± 0.54	0.42 ± 0.47	0.39 ± 0.43	0.38 ± 0.42	0.51 ± 0.50	0.44 ± 0.48
Android Subcutaneous Fat Mass (kg)	1.14 ± 0.68	1.06 ± 0.57	1.09 ± 0.53	1.09 ± 0.52	1.12 ± 0.49	1.10 ± 0.54	1.64 ± 0.94	1.45 ± 0.80	1.36 ± 0.72	1.34 ± 0.71	1.44 ± 0.72	1.44 ± 0.78
Gynoid Fat Mass (kg)	3.53 ± 1.54	3.26 ± 1.25	3.36 ± 1.19	3.42 ± 1.20	3.61 ± 1.18	3.42 ± 1.25	4.93 ± 1.81	4.59 ± 1.60	4.44 ± 1.42	4.33 ± 1.40	4.47 ± 1.45	4.54 ± 1.55

ANOVA showed that VFM was significantly higher in males and females consuming larger amounts of alcohol (Q4, *p* **<** 0.001). Table 1 shows that the mean TFM and VFM were higher in non-drinkers compared with low-to-moderate drinkers (Q1-3) in both sexes. However, Q4 drinkers had higher VFM and TFM compared to non-drinkers in males, and similar VFM in females. The collinearity of VFM and TFM required an approach to study the change in the proportion of VFM relative to TFM across consumption quartiles, hence we used %VFM.

**Fig. 1:**
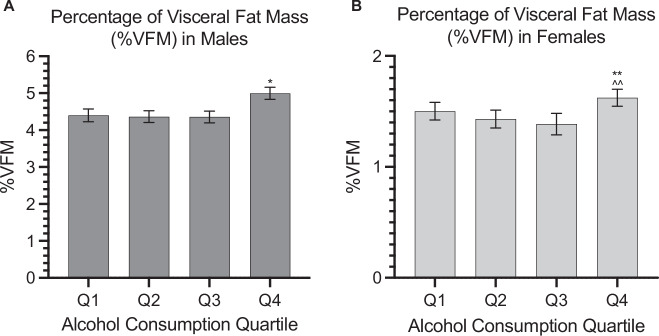
Percentage of visceral fat mass to total body fat mass in quartiles of alcohol consumption in men and women. Results of ANCOVA to analyse for significant differences in the adjusted %VFM (mean + 95% CI) in males (**A**) and females (**B**) across alcohol consumption quartiles. Male adjusted means: Q1 (4.401), Q2 (4.365), Q3 (4.515), Q4 (4.997). Female adjusted means: Q1 (1.502), Q2 (1.431), Q3 (1.386), Q4 (1.623). *=significantly different to Q1–3 (*p* *<* 0.001); **=significantly different to Q3 (*p* *<* 0.001); ^^=significantly different to Q2 (*p* *=* 0.005).

We used ANCOVA to analyse the quartile-specific associations between alcohol consumption and %VFM, adjusting for confounders. Overall, %VFM was higher in males. Among both sexes, adjusted mean %VFM was significantly higher in Q4. In males, %VFM was 10.7% higher in Q4 compared to Q3, and 13.5% higher in Q4 than Q1 (*p* < 0.001) with no significant differences across Q1–3. In females, %VFM was significantly higher in Q4 than Q2-3 (*p* = 0.005) but non-significant for Q1, despite an 8.1% relative difference. Similar to males, the steepest rise in %VFM occurred between Q3 and Q4, with a 17.1% higher value in Q4 (*p* < 0.001). These findings suggest that heavy alcohol consumption is associated with a disproportionate accumulation of visceral fat relative to total fat, as illustrated in Fig. 1A (males) and Fig. 1B (females).

A supplementary interaction model identified significant differences in the relationship of VFM-TFM across alcohol groups in males (p = 0.028) and females (p < 0.001). In both sexes, non-drinkers and light-to-moderate drinkers (Q1-3) showed similar, flatter VFM-TFM slopes, whereas Q4 exhibited a markedly steeper slope, consistent with the disproportionately greater VFM in the highest alcohol consumption group observed in the previous ANCOVA analysis. This is visualised in the Supplementary Figure.

The categorical nature of the quartiles requires support from a more innately dose-dependent assessment. Hence, linear regression was used to further examine the relationship between alcohol consumption and VFM and its confounders. In univariate analysis, there is a strong positive association between alcohol consumption and VFM in both males (ß=1.337 (1.205-1.483), p < 0.001) and females (ß=1.132 (1.033-1.245), p = 0.009). This remained significant after adjustment for all confounders in males (ß=1.104 (1.040-1.167), p < 0.001) and females (ß=1.102 (1.029-1.180), p = 0.006). For comparison, similar regression models tested for an association between alcohol and waist circumference. In females, there was no significant association, before or after confounder adjustment. However, in males, a statistically significant relationship was observed in univariate (ß=1.247 (1.127-1.380), p < 0.001) and multivariate regression (ß=1.054 (1.019-1.091), p = 0.009).

## Discussion

The aim of this study was to model the association between weekly alcohol intake and precisely-measured VFM. We provided an accurate, sex-specific analysis of this relationship in a very large, population-based sample.

Additional analyses showed a weaker association between alcohol consumption and waist circumference, suggesting that conventional anthropometric measures of abdominal adiposity lack sensitivity to detect alcohol-related changes in VFM, reinforcing the need for precise VFM-quantification.

Interestingly, non-drinkers had higher VFM and BMI than drinkers across all quartiles in females and Q1-3 in males. This pattern, reported previously, likely reflects the heterogeneity among non-drinkers (absence of data on previous alcohol consumption, and complex interactions of other lifestyle habits) [20].

This study is limited by a lack of granular information on possible confounders such as education, dietary information, and type of alcoholic beverage. Its cross-sectional design and reliance on self-reported data also preclude causal inference.

Consequently, prospective approaches to studying this relationship are warranted, alongside mechanistic studies using alcohol interventions to understand adipose tissue function. Finally, DXA only quantifies VFM within the android rectangle, potentially underestimating the effect of alcohol consumption on total VFM.

In conclusion, we demonstrate a positive dose-response relationship between alcohol consumption and VFM in both sexes. The highest alcohol consumption group displays over a 10% higher proportional VFM, independent of TFM, indicating a preferential accumulation of visceral fat over total fat among men and women consuming more than 17 and 10 units per week, respectively.

## Supplementary information

Supplementary figure

## Data Availability

The datasets generated during and/or analysed during the current study are available from the corresponding author on reasonable request.
