# Supplementary material for: Greater visceral fat mass accumulation with high alcohol consumption
Source: Int J Obes (Lond). 2026 Feb 25;50(6):1360–3. doi: 10.1038/s41366-026-02030-5 (PMC13287011; doi:10.1038/s41366-026-02030-5)
Supplement: Supplementary file 1 — Supplementary figure [file 41366_2026_2030_MOESM1_ESM.pdf]

Supplementary Figure – Regression slopes of TFM versus VFM in males (A) and females (B) across non-drinkers and alcohol consumption quartiles (Q1-4). The interaction term used was VFM ~ TFM x alcohol quartile. Male slope coefficients: Non-drinkers(0.076), Q1(0.076), Q2(0.077), Q3(0.077), Q4(0.084*). Female slope coefficients: Non-drinkers(0.038), Q1(0.039), Q2(0.040), Q3(0.038), Q4(0.044**). *=p=0.028, **=p<0.001.

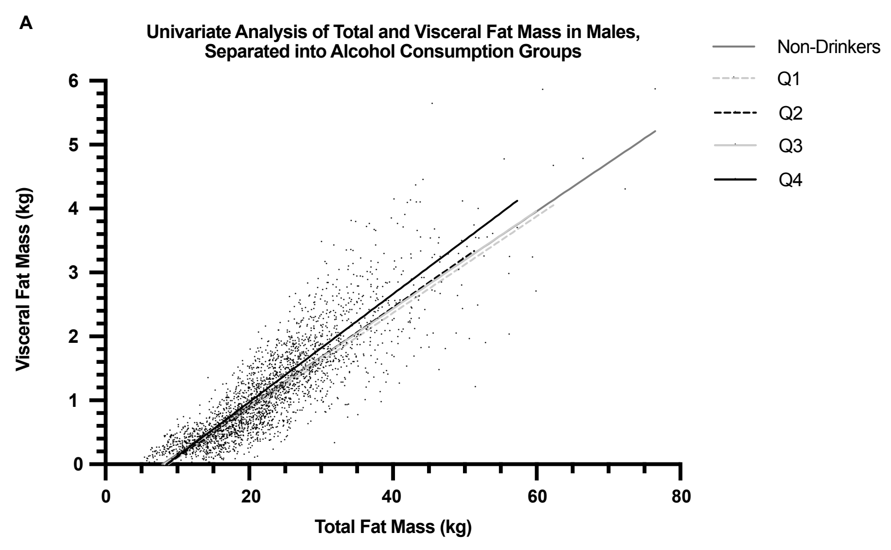

A
Univariate Analysis of Total and Visceral Fat Mass in Males, Separated into Alcohol Consumption Groups
Non-Drinkers
Q1
Q2
Q3
Q4
Visceral Fat Mass (kg)
0
1
2
3
4
5
6
Total Fat Mass (kg)
0
20
40
60
80

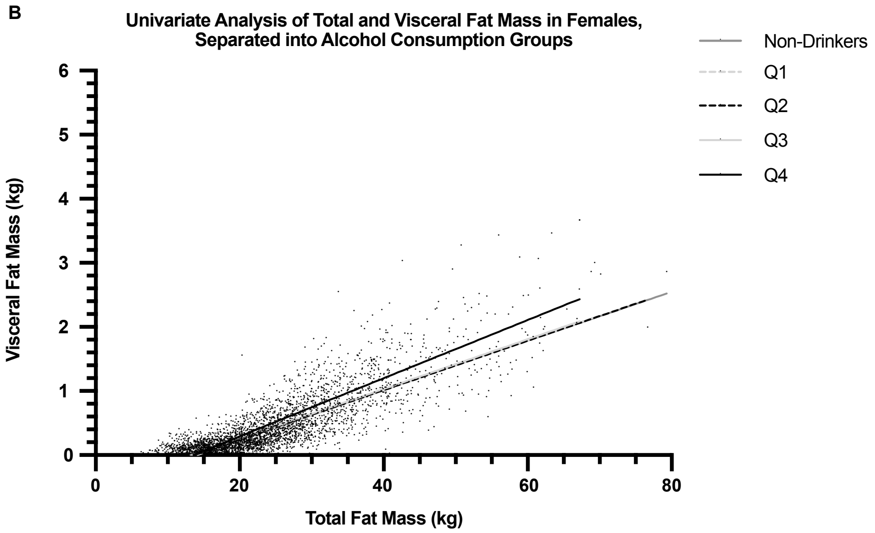
B
Univariate Analysis of Total and Visceral Fat Mass in Females, Separated into Alcohol Consumption Groups
Non-Drinkers
Q1
Q2
Q3
Q4
Visceral Fat Mass (kg)
Total Fat Mass (kg)
0
1
2
3
4
5
6
0
20
40
60
80
